# Young age and high BMI are predictors of early revision surgery after primary anterior cruciate ligament reconstruction: a cohort study from the Swedish and Norwegian knee ligament registries based on 30,747 patients

**DOI:** 10.1007/s00167-019-05487-2

**Published:** 2019-03-16

**Authors:** Thorkell Snaebjörnsson, Eleonor Svantesson, David Sundemo, Olof Westin, Mikael Sansone, Lars Engebretsen, Eric Hamrin-Senorski

**Affiliations:** 1 Department of Orthopaedics, Institute of Clinical Sciences, The Sahlgrenska Academy, University of Gothenburg, Gothenburg, Sweden; 2 Department of Orthopaedics, Sahlgrenska University Hospital, Mölndal, Sweden; 3 Department of Health and Rehabilitation, Institute of Neuroscience and Physiology, The Sahlgrenska Academy, University of Gothenburg, Gothenburg, Sweden; 4 University of Oslo, Oslo University Hospital, Oslo, Norway

**Keywords:** Anterior cruciate ligament, Reconstruction, Revision, Autograft, Registry, Gender, Body height, Body weight, Body mass index, Smoking, Smokeless tobacco, Graft failure

## Abstract

**Purpose:**

To analyse patient-related risk factors for 2-year ACL revision after primary reconstruction. The hypothesis was that younger athletes would have a higher incidence of an early ACL revision.

**Methods:**

This prospective cohort study was based on data from the Norwegian and Swedish National Knee Ligament Registries and included patients who underwent primary ACL reconstruction from 2004 to 2014. The primary end-point was the 2-year incidence of ACL revision. The impact of activity at the time of injury, patient sex, age, height, weight, BMI, and tobacco usage on the incidence of early ACL revision were described by relative risks (RR) with 95% confidence intervals (CI).

**Results:**

A total of 58,692 patients were evaluated for eligibility and 30,591 patients were included in the study. The mean incidence of ACL revision within 2 years was 2.82% (95% CI 2.64–3.02%). Young age (13–19) was associated with an increased risk of early ACL revision (males RR = 1.54 [95% CI 1.27–1.86] *p* < 0.001 and females RR = 1.58 [95% CI 1.28–1.96] *p* < 0.001). Females over 1 SD in weight ran an increased risk of early ACL revision (RR = 1.82, [95% CI 1.15–2.88] *p* = 0.0099). Individuals with a BMI of over 25 ran an increased risk of early ACL revision (males: RR = 1.78, [95% CI 1.38–2.30] *p* < 0.001 and females: RR = 1.84, [95% CI 1.29–2.63] *p* = 0.008).

**Conclusion:**

Young age, a BMI over 25, and overweight females were risk factors for an early ACL revision.

**Level of evidence:**

II.

## Introduction

Graft failure after surgical reconstruction of the anterior cruciate ligament (ACL) is a known complication. The previous studies have identified patient age [1–4], tobacco use [5], and participation in sports at competitive level [4, 6, 7] as risk factors for revision ACL reconstruction. Data from the Scandinavian registries have previously been used in similar studies [8, 9] and these data are a valuable asset in identifying risk factors for revision surgery [10, 11].

Patients sustaining an ACL injury after playing football [9, 12] at a young age have been shown to run a higher risk of early ACL revision after primary reconstruction, while participation in other sports has not been as strongly associated with an ACL injury [13]. A large proportion of reruptures are known to occur within 2 years of the primary reconstruction [14, 15]. It has, therefore, been suggested that return to sport should be delayed until 12–24 months after the primary ACL reconstruction [16].

Increased knowledge of how patient-related factors impact on the risk of ACL revision is important to be able to individualise treatment and target modifiable factors associated with increased risk.

In this prospective cohort study, we aimed to determine the effect of patient-related variables (activity at the time of injury, age, patient gender, height, weight and body mass index (BMI), smoking, and the usage of smokeless tobacco) on revision ACL reconstruction within 2 years of the primary ACL reconstruction. The hypothesis of this study was that younger patients would have a higher incidence of early ACL revision.

## Materials and methods

Data were extracted from the Swedish National Knee Ligament Registry (SNKLR) and the Norwegian Knee Ligament Registry (NKLR). Patients eligible for inclusion were registered for primary ACL reconstruction from 2004 (Norway) and 2005 (Sweden) to 31 December 2014. The inclusion criteria were age between 13 and 59 years of age at the reconstruction, and ACL reconstruction performed with hamstring tendon (HT) or patella tendon (PT) autografts. All patients were followed for 2 years. Patients who underwent contralateral ACL reconstruction during the study period or had sustained concomitant bone, vascular or other ligament damage were excluded from the study.

### The Norwegian and Swedish National Knee Ligament Registries

The NKLR was initiated in 2004 and the SNKLR in 2005 [17]. Data are collected prospectively and the estimated coverage of primary ACL reconstructions in the registries is close to 90% [18] in Sweden and 86% [19] in Norway. Surgical data, including primary and revision ACL reconstruction, are registered by the treating surgeon. Patients are identified by their unique social security number [20]. Follow-up using the Knee injury and Osteoarthritis Outcome Score (KOOS) is carried out by both registries. This is done preoperatively and, in Norway 2-, 5-, and 10-year postoperatively, while, in Sweden, follow-up takes place 1-, 2-, 5-, and 10-year postoperatively [17, 18, 21].

### Variables

The current study investigated the following eight variables: sports activity at the time of primary ACL injury, patient sex, patient age, body height, body weight, BMI, cigarette smoking, and the usage of smokeless tobacco. In the SNKLR and NKLR, data on activity at the time of injury are available. Body mass index was categorised as underweight (< 18.5 kg/m^2^), normal range (18.5–24.9 kg/m^2^), overweight (25.0–29.9 kg/m^2^), and obese (≥ 30.0 kg/m^2^), according to the World Health Organization [22]. A statistical analysis of BMI was adjusted by dividing the group in two; one group of patients who were 20 years of age or older, and one with patients 19 years of age or younger. This was done because of extensive changes in BMI from birth through early adulthood [23]. Body height and body weight were stratified according to the central limit theorem and divided into sexes. Data analyses for demographics and anthropometry were made separately for men and women.

### Outcome measurements

The primary end-point in this study was the 2-year cumulative incidence of ACL revision surgery, defined as the occurrence of new ipsilateral ACL reconstruction within 2 years of the primary ACL reconstruction. Follow-up began on the same day as the primary ACL reconstruction was performed and the end-point was either 2 years of follow-up or revision surgery, whichever occurred first.

The regional ethical review board in Stockholm, Sweden, approved the research (ID 2011/337-31/3).

In Norway, informed consent is obtained for all patients [21].

During the study period, access was bound to unidentifiable data from the database. Data from the NKLR were treated according to Norwegian legislation [21].

### Statistical analysis

Data files from the SNKLR and NKLR were combined and statistical analyses were performed using the SAS System for Windows, version 9; SAS Institute, Cary, North Carolina, USA.

Descriptive statistics for patient demographics were reported as numbers and percentages for categorical variables.

Continuous variables were reported as the mean, standard deviation, median, minimum, and maximum.

The impact of activity at the time of injury, age, patient sex, height, weight and BMI, smoking, and the usage of smokeless tobacco on the incidence of ACL revision was reported as relative risks (RR) with 95% confidence intervals (CI) estimated using generalised linear models with binomial distribution and log-link function. Adjustments for known confounders were made using multivariable analysis; adjusting for age, graft diameter, graft type, fixation in the tibia and femur, meniscal injury and cartilage injury, time to surgery, and country. All tests were two-sided and conducted at the 5% significance level. Statistical significance was defined as a 95% CI for risk estimates not including 1.00 and *p* < 0.05.

## Results

**Fig. 1:**
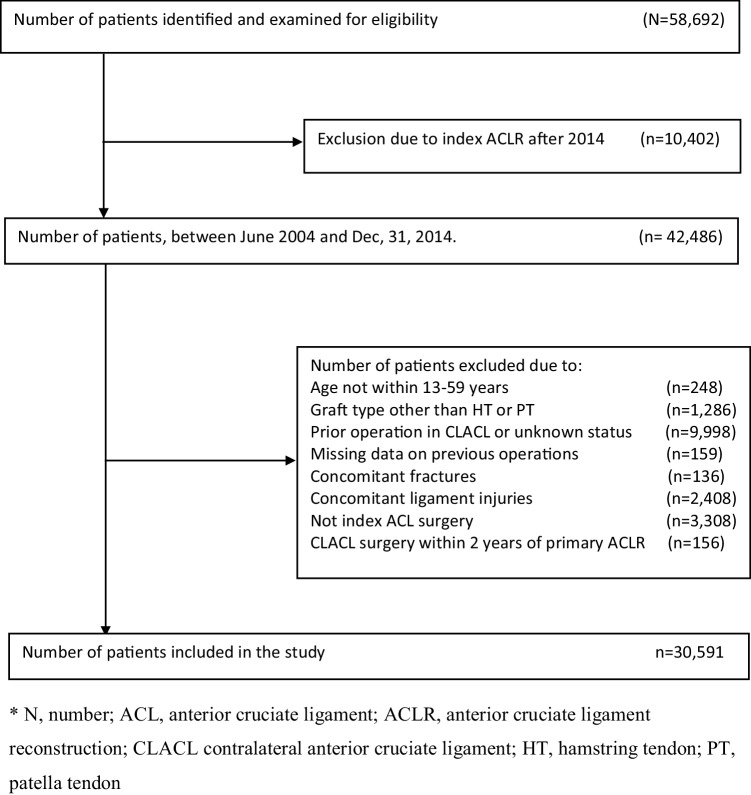
Flow chart of inclusion and exclusion criteria

A total of 58,692 individuals were identified in the SNKLR and NKRL during the study period and examined for eligibility (Fig. 1). A total of 30,591 individuals (17,446 males and 13,145 females) were included (Table 1). A total of 20,546 patients originated from Sweden, while 10,045 came from Norway. A total of 864 individuals had undergone ACL revision within 2 years of the primary ACL reconstruction, corresponding to a crude revision rate of 2.82%.

**Table 1:** Baseline demographics

	Total (*n* = 30,591)	Males (*n* = 17,446)	Females (*n* = 13,145)
Age at index ACL injury	24.4 (9.2)	25.2 (8.5)	23.2 (9.9)
Age at index ACLR	26.5 (9.7)	27.5 (9.0)	25.1 (10.4)
Adolescents	9430 (30.8%)	3755 (21.5%)	5675 (43.2%)
Height	174.7 (9.1)	180.5 (6.6)	167.6 (6.1)
Weight	75.6 (14.2)	82.5 (12.1)	67.1 (11.8)
Body mass index	24.7 (3.6)	25.3 (3.3)	23.9 (3.9)
Smokers	1732 (11.7%)	1003 (12.3%)	729 (11.0%)
Smokeless tobacco users	1820 (15.0%)	1530 (22.9%)	290 (5.3%)
HT autograft	26,537 (86.7%)	15,039 (86.2%)	11,498 (87.5%)
Surgery duration	74.5 (24.2)	75.1 (24.3)	73.6 (24.0)
Injury-to-surgery (months)	17.2 (31.0)	17.3 (30.4)	17.1 (31.9)
Meniscal injury	10,958 (42.4%)	6755 (44.8%)	4203 (39.1%)
Cartilage injury	7033 (27.2%)	4441 (29.5%)	2592 (24.1%)
Activity			
Soccer/football	13,673 (44.9%)	9172 (52.8%)	4501 (34.4%)
Floorball	1916 (6.3%)	1203 (6.9%)	713 (5.5%)
Handball	2718 (8.95%)	598 (3.4%)	2120 (16.2%)
Alpine skiing	2435 (8.0%)	1036 (6.0%)	1399 (10.7%)

For categorical variables *n* (%) is presented, while, for continuous variables, the mean (SD) is presented

*ACL* anterior cruciate ligament, *ACLR* anterior cruciate ligament reconstruction, *HT* hamstring tendon, adolescents, 13–19 years of age

### Activity at the time of the injury

A total of 13,673 patients played football at the time of injury. This activity was the most common sport for men (52.8%), while floorball (6.9%) was second (Table 2). Males playing floorball at the time of the ACL injury ran a significantly lower risk of early ACL revision compared with all other sports. For women, football (34.4%) was also the most common activity, with handball (16.2%) being second (Table 2). Females who sustained an ACL injury, while downhill skiing ran a significantly lower risk of early ACL revision when compared with females participating in other sports when the ACL injury occurred.

**Table 2:** Incidence of revision surgery within 2 years, activity at the time of ACL injury

Activity	Men				Women			
	Incidence (%)	Adjusted RR	95% CI	*p* value	Incidence (%)	Adjusted RR	95% CI	*p* value
Football	3.07	1.09	0.91–1.30	n.s.	2.98	0.95	0.76–1.19	n.s.
Floorball	1.25	0.47	0.28–0.79	0.0048	2.24	0.81	0.52–1.41	n.s.
Skiing^a^	1.54	0.67	0.41–1.09	n.s.	0.71	0.33	0.17–0.60	0.0005
Handball	4.01	1.15	0.78–1.70	n.s.	4.1	1.25	0.97–1.60	n.s.

Adjusted for age, time to surgery, graft type, graft diameter, femoral fixation (cortical, cross/rigid, metal screw, bioabsorbable screw), tibial fixation (post, bioabsorbable screw), concomitant meniscal injury, concomitant cartilage injury, and country

*ACL* anterior cruciate ligament, *RR* relative risk, *CI* confidence interval

^a^Downhill skiing

### Patient sex

There was no significant difference between men and women in the risk of early ACL revision (Table 3).

**Table 3:** Revision surgery within 2 years, men versus women

Activity	Incidence %	Unadjusted			Adjusted^a^		
		RR	95% CI	*p* value	RR	95% CI	*p* value
Patient sex	2.83 versus 2.82	1	0.88–1.14	n.s.	1.13	0.98–1.29	n.s.

*RR* relative risk, *CI* confidence interval

^a^Adjusted for age, time to surgery, graft type, graft diameter, femoral fixation, tibia fixation, concomitant meniscal injury, concomitant cartilage injury, and country

### Age

The study participants were stratified into three different age groups (Table 4). For males, the adolescents (13–19 years of age) had significantly increased risk of ACL revision within 2 years of the primary reconstruction compared with all the others, while the oldest age group ran a reduced risk of early ACL revision compared with all other age groups. Within the female group, the youngest age group ran an increased risk of compared with all older females. Both the other age groups, 20–29 and 30–59 years of age, ran a reduced risk of revision when compared with all the other female age groups.

**Table 4:** Incidence of revision surgery within 2 years, age at the time of ACL reconstruction

Age	Men				Women			
	Incidence (%)	Adjusted RR	95% CI	*p* value	Incidence	Adjusted RR	95% CI	*p* value
13–19	3.97	1.52	1.26–1.84	< 0.001	3.65	1.58	1.28–1.95	< 0.001
20–29	2.8	1	0.84–1.20	n.s.	2.25	0.73	0.57–0.93	0.012
30–59	2.17	0.66	0.54–0.81	< 0.001	2.14	0.75	0.58–0.97	0.030

Adjusted for time to surgery, graft type, graft diameter, femoral fixation, tibia fixation, concomitant meniscal injury, concomitant cartilage injury, and country

*ACL* anterior cruciate ligament, *RR* relative risk, *CI* confidence interval

### Anthropometric data

No significant differences in early ACL revision were found when analysing the effect of patient height (Table 5). For women weighing more than one standard deviation over the mean weight (Table 6), there was an increased risk of early ACL revision when compared with all other women.

**Table 5:** Incidence of revision surgery within 2 years, height

Height	Men				Women			
	Incidence (%)	Adjusted RR	95% CI	*p* value	Incidence (%)	Adjusted RR	95% CI	*p* value
< − 1 SD	2.09	1.1	0.69–1.76	n.s.	2.18	0.91	0.56–1.49	n.s.
± 1 SD	1.82	0.82	0.57–1.18	n.s.	2.25	0.84	0.59–1.19	n.s.
> 1 SD	2.35	1.27	0.80–2.00	n.s.	3.21	1.4	0.93–2.11	n.s.

Adjusted for age, graft diameter, femoral fixation, country

*RR* relative risk, *CI* confidence interval, *SD* standard deviation

**Table 6:** Incidence of revision surgery within 2 years, weight

Weight	Men				Women			
	Incidence (%)	Adjusted^a^ RR	95% CI	*p* value	Incidence (%)	Adjusted^a^ RR	95% CI	*p* value
< − 1 SD	2.49	1.09	0.69–1.73	n.s.	2.03	0.77	0.43–1.36	n.s.
± 1 SD	1.89	0.86	0.59–1.26	n.s.	2.29	0.8	0.54–1.17	n.s.
> 1 SD	1.83	1.21	0.70–2.11	n.s.	3.3	1.82	1.16–2.88	0.0099

*RR* relative risk, *CI* confidence interval, *SD* standard deviation

^a^Adjusted for age, time to surgery, graft type, graft diameter, femoral fixation, concomitant meniscus injury, concomitant cartilage injury, and country

When combining height and weight in BMI for patients 20 years of age or older (Table 7), the results were similar for both men and women. Participants with a BMI of under 25 ran a significantly lower risk of early ACL revision compared with the rest of the study group. Participants with a BMI of 25 or more ran an increased risk of an early ACL revision compared with patients with a BMI of under 25. More specifically, men with a BMI of between 25 and 30 ran an increased risk of early ACL revision when compared with all other men. Women with a BMI of between 25 and 30 also ran an increased risk of ACL revision when compared with all other women. Patients with a BMI of over 30 did not show any increased risk of early ACL revision compared with all other groups.

**Table 7:** Incidence of revision surgery within 2 years, adults BMI

BMI	Men				Women			
	Incidence (%)	Adjusted RR^a^	95% CI	*p* value	Incidence (%)	Adjusted RR^a^	95% CI	*p* value
18.5 < 25	2.24	0.57	0.44–0.73	< 0.001	1.95	0.55	0.38–0.78	0.009
≥ 25	4.21	1.74	1.35–2.24	< 0.001	3.94	1.84	1.29–2.63	0.008
25 to < 30	4.75	1.95	1.52–2.50	< 0.001	4.37	1.90	1.33–2.71	0.004
≥ 30	1.34	0.53	0.28–1.03	n.s.	2.45	1	0.51–1.95	n.s.

Adults, aged 20 and above included

*RR* relative risk, *CI* confidence interval

^a^Adjusted for age, time to surgery, graft type, graft diameter, femoral fixation, tibia fixation, concomitant meniscal injury, concomitant cartilage injury, and country

The effect of BMI on the risk of ACL revision for patients 19 years of age or younger (Table 8) was similar to older patients. For men, those with a BMI of between 25 and 30 ran an increased risk of revision compared with all other men. All women 19 years of age or younger with a BMI of over 25 ran an increased risk of revision compared with all other women.

**Table 8:** Incidence of revision surgery within 2 years, BMI adolescents

BMI	Men				Women			
	Incidence (%)	Adjusted RR	95% CI	*p* value	Incidence (%)	Adjusted RR	95% CI	*p* value
18.5 < 25	3.74	0.66	0.44–0.99	0.045	3.45	0.55	0.40–0.77	0.0004
≥ 25	5.62	1.56	1.05–2.34	0.030	5.68	1.77	1.29–2.52	0.0006
25 to < 30	6.57	1.92	1.28–2.87	0.0015	6.29	1.99	1.45–2.86	< 0.001
≥ 30					1.56	0.43	0.10–1.67	n.s.

Adjusted for age, time to surgery, graft type, graft diameter, femoral fixation, tibia fixation, concomitant meniscal injury, concomitant cartilage injury, and country

Adolescents, aged 19 and below included

*RR* relative risk, *CI* confidence interval;

### Tobacco use

The use of either tobacco or smokeless tobacco for patients undergoing primary ACL reconstruction was not a risk factor for early ACL revision (Table 9) when compared with patients not using tobacco or smokeless tobacco.

**Table 9:** Incidence of revision surgery within 2 years, tobacco use

Tobacco use	Men				Women			
	Incidence (%)	Adjusted RR	95% CI	*p* value	Incidence (%)	Adjusted RR	95% CI	*p* value
Smoking	1.2	0.73	0.40–1.36	n.s.	1.92	1.49	0.83–2.65	n.s.
Non-smoking	2.42	1.28	0.87–1.87	n.s.	2.07	0.99	0.44–2.22	n.s.

Adjusted for age, time to surgery, graft type, graft diameter, femoral fixation, concomitant meniscal injury, concomitant cartilage injury, and country

*RR* relative risk, *CI* confidence interval

### High-risk individuals

A further analysis was performed for adolescents who played football at the time of the injury. Young football players ran a significantly increased risk of early ACL revision (Table 10) among both men and women.

**Table 10:** Incidence of revision surgery within 2 years, high-risk individuals

Activity	Men				Women			
	Incidence (%)	Adjusted^a^ RR	95% CI	*p* value	Incidence (%)	Adjusted^a^ RR	95% CI	*p* value
Risk group	4.36	1.55	1.25–1.92	< 0.001	3.96	1.49	1.18–1.87	0.0007

*RR* relative risk, *CI* confidence interval

^a^Adjusted for time to surgery, graft type, graft diameter, femoral fixation, tibia fixation, concomitant meniscal injury, concomitant cartilage injury, and country

## Discussion

The most important finding in this study was that adolescents run an increased risk of early ACL revision. Female downhill skiers and male floorball players ran an increased risk of early ACL revision compared with other sports. Individuals with a BMI at either end (18 < 25 and ≥ 30) of the BMI scale ran a lower risk of early ACL revision compared with those with a BMI of between 25 and 30. Adolescents playing football at the time of primary ACL injury ran a significantly increased risk of early ACL reconstruction among both men and women.

In the present study, playing floorball at the time of the ACL injury significantly decreased the risk of an early ACL revision in men compared with all other sporting and non-sporting activities at the time of the index injury. Floorball is played professionally in Sweden and, in a recent Swedish epidemiological study, [24] ACL injuries were much more frequent in women. For women, sustaining an ACL injury during downhill skiing reduced the risk of an early ACL revision when compared with all other activities at the time of the index injury. Interestingly, playing football was the most common activity that leads to injury for both men and women. This is in accordance with the previous studies in the field [17]. Unfortunately, no data are available about return to sport or the age or level of individuals suffering from ACL injuries in Scandinavian registries, on which the current study is based on. The type of sport and activity at the time of injury could have a significant impact on the incidence of early ACL revision, with a recent large-scale emphasis on appropriate readiness to return to sport criteria [16, 25].

### Patient gender

Women are known to run a greater risk of ACL injuries compared with males, [26] with sex-related differences in knee and ankle kinematics [27] or even the timing of knee forces [28] as a possible explanation. Studies have been unable to identify any significant sex-specific difference in the incidence of graft failure after ACL reconstruction [29, 30]. The results of the present study further underline that there is no apparent sex-specific difference related to the incidence of early ACL revision.

### Age

Adolescents (13–19 years of age) ran a significantly higher risk of early ACL revision compared with older age groups. Older women (both 20–29 and 30–59) ran a significantly reduced risk of revision compared with other women. Men between 30 and 59 years of age ran a significantly lower risk of early ACL revision compared with younger men.

These results are in line with multiple other studies indicating an increased risk of reinjury or revision ACL surgery for younger patients [7, 9, 31–34]. This can be partly explained by the higher activity level of younger athletes, returning early after rehabilitation [35], insufficient rehabilitation or the fact that a repeated ACL surgery after initial failure can be a less favorable alternative for older patients who are not attempting to return to their preinjury activity level.

### Anthropometric data

The body weight was only found to be predictive for women who had more than 1 SD over the average weight [67.1 (SD 11.8)] and BMI was a predictor of ACL revision. The results indicate that, when overweight (25–30 kg/m^2^) and obese (> 30 kg/m^2^) patients are treated as one group, the patients run an increased risk of ACL revision. Similar results were found for women, 19 years of age or younger, and older patients of both sex. When dividing patients with a BMI of over 25 into two different groups, it is only the overweight group (BMI 25–30) and not the obese group (BMI ≥ 30) that runs a significantly increased risk of revision surgery for all age groups. Biomechanical differences in terms of muscle control for patient groups differing in BMI [36] may play a part as well as the patients’ level of participation in sport. Prior studies are inconclusive when it comes to the effect of BMI on ACL revision [37–39].

### Tobacco

Tobacco use was not a risk factor for early ACL revision surgery. Patients using tobacco have significantly poorer subjective outcome measurements, as well as an increased risk of adverse events such as infection and VTE after ACL reconstruction, [5, 40], although the results have been inconclusive in terms of the risk of reinjury and subsequent ACL revision.

### High-risk individuals

Football players were analysed further, because this is the largest subgroup of patients in the present study and adolescents run the highest risk of early ACL injury compared with older age groups. It is known that younger players and men [41] have greater success in terms of their ability to return to sport and football after ACL reconstruction [12, 25, 42]. The current results indicate a similar incidence of early ACL revision surgery for men compared with women among these high-risk individuals. It is noteworthy that previous studies [18] have specifically identified young female football players at increased risk of ACL revision surgery, which conflicts with the findings in this study.

### Limitations

In the present study, only ACL revision was studied as an outcome. However, this underestimates the true occurrence of graft failure after primary reconstruction. Since the SNKLR and NKLR only register revision surgery and not graft failure, it was not possible to identify all patients suffering from graft failure. Patients with graft failure may choose not to undergo ACL revision for a number of reasons. This means that there is a possibility of different predictors of ACL revisions than of graft failures.

Bearing in mind the strict criteria for the end-point, we have avoided a discussion of how to confirm clinical graft failure, thereby making this study easier to compare and replicate.

## Conclusion

Younger age, overweight women, and patients with BMI between 25 and 30 all run an increased risk of early ACL revision surgery after primary ACL reconstruction. Patient sex, tobacco use, and height were not shown to be significant risk factors for early ACL revision. Men playing floorball and women sustaining their ACL injury during downhill skiing run a reduced risk of early ACL revision compared with patients performing other activities at the time of injury.

Playing football and adolescence at the time of the primary ACL injury results in an increased risk of early ACL revision among both men and women.
